# Elucidating some common biases in randomized controlled trials using directed acyclic graphs

**DOI:** 10.1007/s10654-025-01298-7

**Published:** 2025-09-11

**Authors:** Erin E. Gabriel, Alex Ocampo, Arvid Sjölander

**Affiliations:** 1 Section of Biostatistics, Department of Public Health, University of Copenhagen, Copenhagen, Denmark; 2 Novartis Pharma AG, Basel, Switzerland; 3 Department of Medical Epidemiology and Biostatistics, Karolinska Institutet, Solna, Sweden; 4 Pioneer Centre for SMARTbiomed, University of Copenhagen, Copenhagen, Denmark

**Keywords:** Blinding, Causal inference, DAGs, Identification, Instrumental variable, ITT

## Abstract

Although the ideal randomized clinical trial is the gold standard for causal inference, real randomized trials often suffer from imperfections that may hamper causal effect estimation. Stating the estimand of interest can help reduce confusion about what is being estimated, but it is often difficult to determine what is and is not identifiable given a trial’s specific imperfections. We demonstrate how directed acyclic graphs can be used to elucidate the consequences of common imperfections, such as noncompliance, unblinding, and drop-out, for the identification of the intention-to-treat effect, the total treatment effect and the physiological treatment effect. We assert that the physiological treatment effect is not identifiable outside a trial with perfect compliance and no dropout, where blinding is perfectly maintained.

**Supplementary Information:**

The online version contains supplementary material available at https://doi.org/10.1007/s10654-025-01298-7.

## Introduction

The ideal randomized controlled trial (RCT) is the gold standard for causal inference. However, real (as opposed to ideal) randomized trials often suffer from imperfections such as noncompliance, unblinding, and participant drop-out. Although it is widely recognized that such complications can be ‘problematic’, their specific consequences are often not clearly articulated. In particular, whether or not they lead to bias and/or non-identifiability generally depends on the target estimand of the trial [1], but this estimand is often not made explicit and related to the aforementioned complications.

There is now a large body of literature on how to use Directed Acyclic Graphs (DAGs) for addressing complications in observational studies, including unmeasured (possibly time-varying) confounding, selection bias and measurement error [2–4]. However, the literature on DAGs for randomized trials is much more sparse. The standard DAG presented in the literature [5] is a simplistic version, which illustrates noncompliance but ignores other complications. A recent exception is Ocampo and Bather [6], who used single-world intervention graphs (SWIGs) to discuss the consequences of intercurrent events in RCTs.

In this paper we use DAGs to discuss the consequences of both noncompliance, unblinding, and participant drop-out, in terms of bias and non-identifiability. We focus on three estimands: the intention-to-treat (ITT) effect, the total treatment effect, and the physiological treatment effect. The ITT effect is the effect of being assigned to treatment. The total treatment effect is the effect of actually taking the treatment, including both the physiological and non-physiological (e.g., behavioral and psychological) effect. We define the total treatment effect later as a contrast between two counterfactual scenarios; everybody is treated vs. everybody is untreated. The physiological treatment effect is the part of the effect that is not mediated through the subject’s and treating clinician’s beliefs about, and expectations on, the treatment taken. Arguably, the purpose of blinding is to enable identification of the physiological treatment effect, since blinding, by definition, removes or at least reduces the potential influence of the treatment on these beliefs and expectations, thereby isolating the physiological response to the treatment. We provide a counterfactual definition of the physiological treatment effect later as a so-called ‘natural direct effect’ [7].

Many clinical trials define the ITT effect as the primary estimand. One possible reason for this is that, in the real world, a clinician cannot force patients to take the treatment, only assign them to it. Thus, it can be argued [8], that the ITT effect measures the ‘real world’ effect of the treatment. However, this argument presumes that the degree of compliance with the assignment in the real world is similar to that in the randomized trial, which may not be the case [9]. The degree of compliance could be higher in the real world after the treatment is approved because an approved and widely available treatment may give patients a strong incentive to comply. However, the degree of compliance could also be lower in the real world, as the incentives for clinicians to urge their patients to follow the treatment protocol may not be as strong in the real world as in a tightly controlled trial. Thus, the ‘real world effect’ may be closer to the total treatment or the ITT effect, depending on the context.

The physiological treatment effect is more stable and generalizable than the ITT effect and total treatment effect, as it is less susceptible to trial-specific imperfections such as noncompliance and unblinding, and independent of patients’ or clinicians’ expectations regarding the treatment. Since these imperfections and expectations can differ significantly across individual trials and between trials and real-world settings, decision makers, such as politicians or clinicians, may prioritize understanding the physiological treatment effect. Moreover, accurately estimating the physiological treatment effect is critical for guiding future advancements in the treatment, particularly for refining chemical or biological components acting through physiological pathways. Nevertheless, the physiological treatment effect is rarely explicitly stated as a target estimand. This may be because, as we will argue, the physiological treatment effect is rarely identifiable without additional assumptions not necessarily implied by the trial design.

## Assumptions and notation

We consider an RCT where the aim is to compare an active treatment of interest with a ‘standard of care’ treatment. For simplicity, we assume that the standard of care is no active treatment, disguised as placebo treatment if the trial is blinded, but we note that in many real trials the standard of care would rather be an established active treatment. We further assume that the treatment, standard or active, is a point treatment administered on a single occasion and that compliance with the assigned treatment level is ‘all or nothing.’ We acknowledge that in many real trials, the treatment is administered repeatedly, and the degree of compliance may be partial and also vary over the course of the trial.

We let *Z* be the treatment that a subject in the trial is randomly assigned to, with *Z=1* for ‘active treatment’ and *Z=0* for ‘no treatment’, and let *X* be the treatment that the subject actually takes, with *X=1* for ‘active treatment’ and *X=0* for ‘no treatment’. With perfect compliance, *X=Z* for all subjects. In some settings, the active treatment of interest is not freely available, so that *Z=0* implies that *X=0*, even in the presence of noncompliance. However, to be general, we allow for the possibility that a subject may take active treatment *X=1* even though they were assigned to no treatment *Z=0*. Let *Y* be the outcome of interest, which we assume is either binary or continuous point outcome, measured at a single occasion after randomization. We will use *U* generically for unmeasured confounders; however, the set of variables affected by *U* differs between different types of trials, as detailed below.

To enable a comparison between unblinded and blinded RCTs, and to distinguish between the physiological treatment effect and the non-physiological treatment effect, it will be useful to let $X_{self}$ denote the treatment that a subject *believes that they took*. Similarly, we let $X_{cln}$ denote the treatment that the (team of) clinician(s) believes that a subject took. If the trial is unblinded, or the trial is blinded but the subject breaks the protocol by not taking what is blindly assigned to them, then we assume that both the subject themselves and the clinician(s) are fully aware of what treatment the subject takes, so that $X_{self}=X_{cln}=X$. In contrast, under successful blinding, $X_{self}$ and $X_{cln}$ are random variables, in the sense that some subjects and clinicians may be more inclined to believe that the active treatment was actually taken, where others may believe placebo/no treatment was taken. When $X_{self}$ and $X_{cln}$ are random variables, we will assume they are unmeasured.

A subject’s beliefs about what treatment they took can always affect their behavior during the trial, and this behavior can in turn affect the outcome. For example, believing one received a vaccine against a disease could make one less careful in high risk situations. One could also imagine settings where there are purely psychological effects of the subject’s belief on the outcome. For example, a subject who firmly believes that they took the active treatment may feel better than a subject who firmly believes the opposite.

A clinician’s belief about how a subject was treated can similarly change their behavior towards the subject. For example, a clinician may be more willing to provide rescue treatments to patients they believe to be taking no treatment than those they believe to be on the active treatment. A clinician’s belief about how a subject was treated can also have a direct effect on the outcome. For example, an outcome diagnosis set by an adjudicating clinician may depend directly on the clinician’s belief about the subject’s treatment.

We further let *S* denote the subject’s physical and mental health status in the intermediate period between the treatment taken and the outcome at the end of the trial, not including the outcome itself. This variable is a complex construct that may include things that might be referred to as ’side-effects’ of the treatment. Thus, *X* could potentially affect *S* in any trial. Regardless of whether the trial is blinded or not, *S* may affect $X_{self}$ and $X_{cln}$, e.g., if the subject starts feeling particularly well in the period after taking the treatment, then both the subject themselves and the clinician may be inclined to believe that the subject took the active treatment. We assume that *S* is at least partly unmeasured. That is, the trial may record some aspects of the subject’s intermediate health status, e.g., through a questionnaire, but a comprehensive assessment of this multidimensional construct would often not be practically feasible.

For pedagogical purposes, we will argue as if *S*, $X_{self}$, and $X_{cln}$ are realized at a single time point between the randomization and the outcome, and that the temporal order is such that *S* happens first, and then $X_{self}$ and $X_{cln}$ happens. We note that in reality, these variables are potentially complex processes in time, which may influence each other, and partially for this reason, we will always consider them unmeasurable.

## Estimands

We use standard counterfactual notation [10–13] to define the ITT effect, the total treatment effect, and the physiological treatment effect. Let *Y*(*z*) be the value of *Y* had, potentially counter to fact, the assigned treatment been set to *Z*. Similarly, let *Y*(*x*) be the outcome *Y* had, potentially counter to fact, the treatment actually taken been set to *x*. The ITT effect is some contrast between $E\{Y(z=1)\}$ and $E\{Y(z=0)\}$, for instance, the difference of means $E\{Y(z=1)\}-E\{Y(z=0)\}$ or the ratio of means $E\{Y(z=1)\}/E\{Y(z=0)\}$. The total treatment effect is similarly some contrast between $E\{Y(x=1)\}$ and $E\{Y(x=0)\}$.

To define the physiological treatment effect, let $X_{self}(x)$ and $X_{cln}(x)$ denote the values of $X_{self}$ and $X_{cln}$ had, potentially counter to fact, the treatment actually taken been set to *x*. Let $Y\{X=x,X_{self}(x'),X_{cln}(x')\}$ denote the value of *Y* had *X* been set to *x*, and $X_{self}$ and $X_{cln}$ been set to whatever value they would have had, had *X* been set to *x'*, where *x* and *x'* are not necessarily equal. We define the physiological treatment effect as the natural direct effect [7] of the treatment actually taken, not mediated through $X_{self}$ and $X_{cln}$, which is some contrast between $E[Y\{x=1,X_{self}(x=0),X_{cln}(x=0)\}]$ and $E[Y\{x=0,X_{self}(x=0),X_{cln}(x=0)\}]$. The reason why we define the physiological treatment effect as a natural direct effect is because this effect is precisely what is produced by a trial under ideal conditions—we discuss this in "Blinded trial" section. We note that, although we set *x=0* in our definition of the physiological treatment effect, all statements hereafter equally apply for *x=1*.

By holding the treatment fixed at *x=0* in the physiological treatment effect, from the perspective of $X_{self}$ and $X_{cln}$, we eliminate all effect mediated through the subject’s and clinician’s beliefs about what treatment was taken. We emphasize that this does not eliminate all of the effect mediated through the subject’s intermediate health status *S*. For instance, weight loss treatments may cause reduced appetite that may cause the subject to make dietary changes and/or cause the clinician to recommend dietary changes, which in turn may affect the outcome; this is by our definition part of the physiological treatment effect. However, if a subject believes (correct or not) they have received weight loss treatment and, *due to that belief*, they make dietary changes, then this effect is not part of the physiological treatment effect, as we define it. We also emphasize that our definition of physiological treatment effect encompasses all possible physiological pathways, not only those that the treatment is intended to target. For instance, suppose that a weight loss treatment is intended to target satiety receptors in the gastrointestinal system. However, as an unintended (and unknown) consequence it also improves psychological mood, thereby reducing weight by increasing the chance that the subject engages in weight-loss inducing exercise. This unintended consequence is then also part of the physiological treatment effect, as we define it.

For simplicity, we focus on mean differences and define

$$\begin{aligned} \theta _{ITT}= & E\{Y(z=1)\}-E\{Y(z=0)\},\\ \theta _{trt}= & E\{Y(x=1)\}-E\{Y(x=0)\}\\ & \text {and}\\ \theta _{phys}= & E[Y\{x=1,X_{self}(x=0),X_{cln}(x=0)\}]\\- & E[Y\{x=0,X_{self}(x=0),X_{cln}(x=0)\}]. \end{aligned}$$

## Summary of identification results

In the following sections, we discuss how the identifiability of $\theta _{ITT}$, $\theta _{trt}$ and $\theta _{phys}$ is affected by non-blinding, noncompliance and drop-out. To be clear, when we refer to an effect as ‘identifiable’, we mean that it can be computed from the observed data distribution. For brevity, the identification results are summarized in Table 1. Specifically, without additional assumptions that are not implied by randomization alone, $\theta _{phys}$ is never identifiable, and none of the estimands is identifiable without additional assumptions in the presence of drop-out. In the following sections we also discuss what additional assumptions one could make to achieve identifiability, and propose alternative inferential strategies, such as sensitivity analysis and bounds, when such identifying assumptions are not plausible.

**Table 1:** Identifiability of target estimands

Blinding	Full compliance	No drop-out	$\theta _{ITT}$	$\theta _{trt}$	$\theta _{phys}$
Yes	Yes	Yes	Yes	Yes	No
No	Yes	Yes	Yes	Yes	No
Yes	No	Yes	Yes	Yes	No
No	No	Yes	Yes	No	No
Yes/no	Yes/no	No	No	No	No

## Perfect compliance and no drop-out

We first consider the ideal setting with perfect compliance and no drop-out, and then discuss the complications induced by noncompliance and drop-out.

### Blinded trial

**Fig. 1:**
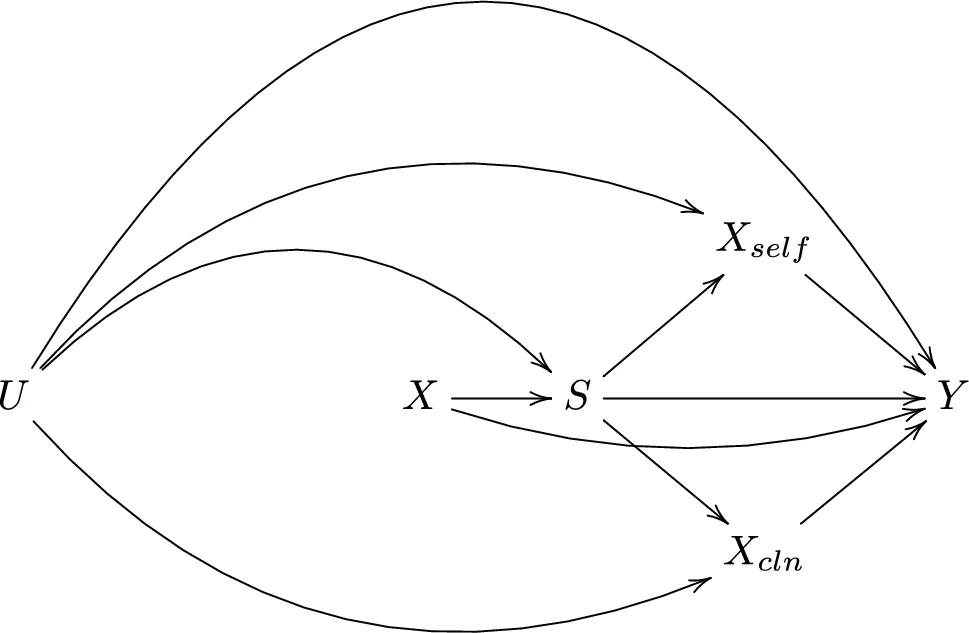
A blinded trial with perfect compliance

The DAG in Fig. 1 represents a blinded trial with perfect compliance and no drop-out. We have omitted *Z* from the DAG since *X* and *Z* are identical, due to perfect compliance. The confounders *U* may affect *S*, $X_{self}$, $X_{cln}$, and *Y*. However, they cannot affect *X*, due to randomization and perfect compliance. The absence of arrows from *X* to $X_{self}$ and $X_{cln}$ follows from triple blinding and perfect compliance. We note that double blinding is generally not sufficient for ruling out these arrows, since it does not guarantee that every single individual (e.g., clinician, statistician) who is involved in the study is blind to the treatment assignment.

The causal paths from *X* to *Y* that do not pass through $X_{self}$ or $X_{cln}$ represent the physiological effect of the treatment on the outcome, whereas the causal paths from *X* to *Y* that pass through $X_{self}$ or $X_{cln}$ represent a non-physiological effect. We emphasize that we here and elsewhere refer to effects on *Y*, not on *S*. In particular, *X* may have a physiological effect on *S*, as represented by the arrow from *X* to *S*, even in the absence of a physiological effect on *Y*, i.e., even if the arrows from *X* to *Y* and from *S* to *Y* are absent.

In this setting, $$\theta _{ITT}=\theta _{trt}$$, and are nonparametrically identifiable as $$ {{E(Y|Z = 1) - E(Y|Z = 0)}} $$$$ {{ = E(Y|X = 1) - E(Y|X = 0)}} $$. Ideally, the blinding entirely removes the influence of the treatment actually taken *X* on the subject’s and clinician’s believes $$X_{self}$$ and $$X_{cln}$$. This could, for instance, happen if the follow-up time of the trial is short, or the mechanism of action of the treatment is very specific to *Y*. In this ideal case, $$\theta _{phys}$$, defined as a natural direct effect, is equal to $$\theta _{ITT}$$ and $$\theta _{trt}$$, and thus also identifiable. In the less ideal case where the blinding does not entirely remove the influence of *X* on $$X_{self}$$ and $$X_{cln}$$, $$\theta _{phys}$$ is not generally identifiable.

### Unblinded trial

**Fig. 2:**
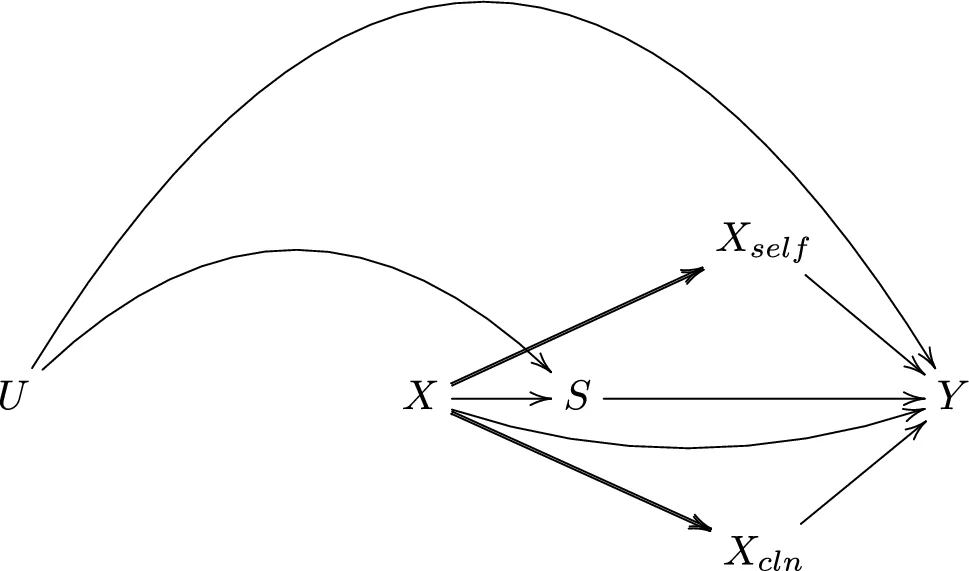
An unblinded trial with perfect compliance

The DAG in Fig. 2 represents an unblinded trial with perfect compliance and no drop-out. Here, we have again omitted *Z*. The bold arrows from *X* to $X_{self}$ and $X_{cln}$ in Fig. 2 represent the deterministic relations $X_{self}=X_{cln}=X$, which follow from unblinding. Due to these deterministic relations, *U* and *S* have no effect on $X_{self}$ and $X_{cln}$.

The path $X\rightarrow X_{self}\rightarrow Y$ includes a purely psychological effect of the treatment. An example was given by FDA ICH Harmonised Tripartite Guideline [14], “Subjects on active drug might report more favorable outcomes because they expect a benefit." Such an effect may be labeled as a ‘placebo effect’, but we note that this term is controversial and refrain from further use.

In this setting, $$\theta _{ITT}=\theta _{trt}$$, and nonparametrically identifiable as $$ {{E(Y|Z = 1) - E(Y|Z = 0) = }} $$$$ {{E(Y|X = 1) - E(Y|X = 0)}} $$. However, $$\theta _{phys}$$ is not identifiable, since an observed association between *X* and *Y*, or between *Z* and *Y*, can be partly or fully explained by the non-physiological pathways from *X* to *Y* that pass through $$X_{self}$$ or $$X_{cln}$$.

We emphasize that, although $\theta _{phys}$ is neither identifiable under the DAG in Fig. 1, nor under the DAG in Fig. 2, the additional assumptions required for identification of $\theta _{phys}$ are substantially more stringent in the latter. When the study is unblinded (Fig. 2), $\theta _{phys}$ is only identifiable if the arrows from $X_{self}$ and $X_{cln}$ to *Y* are assumed absent. In contrast, under blinding (Fig. 1), $\theta _{phys}$ is identifiable if these arrows are assumed absent, but is also identifiable if the arrows from *S* to $X_{self}$ and $X_{cln}$ are assumed absent, or if the arrow from *X* to *S* is assumed absent. In reality, one would perhaps not expect any of these arrows to be entirely absent, but be inclined to believe that $\theta _{phys}$ is at least closer to $\theta _{ITT}$ in the blinded trial than in the unblinded trial.

## Noncompliance but no drop-out

### The ‘standard DAG’ for noncompliance

**Fig. 3:**
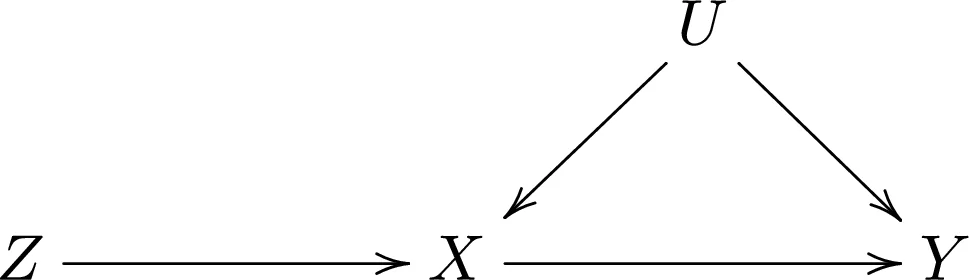
The ‘standard DAG’ for noncompliance

Figure 3 shows the DAG that is typically used in the literature to illustrate an RCT with possible noncompliance [5]. This DAG is simplistic, in the sense that it ignores other complications, such as non-blinding and drop-out.

Under the DAG in Fig. 3, the assigned treatment is a valid instrumental variable (IV). Specifically, *Z* satisfies the three IV assumptions:

1. The assigned treatment is associated with the treatment actually taken.
2. The assigned treatment is independent of the confounders for the treatment actually taken and the outcome.
3. All effect of the assigned treatment on the outcome is mediated through the treatment actually taken.

Under the DAG in Fig. 3, an observed association between *Z* and *Y* can only be explained by the path $Z\rightarrow X\rightarrow Y$, since the path $Z\rightarrow X\leftarrow U\rightarrow Y$ is blocked at the collider *X*. Hence, a non-zero ITT effect implies that *X* has an effect on *Y*.

Notably though, a non-zero ITT effect only implies that *X* has an effect on *Y* *for some subjects*. If there is strong effect heterogeneity, e.g., if the treatment effect is positive for some subjects and negative for other, then, as demonstrated by Balke and Pearl [5], these effects may cancel out so that the total treatment effect is null, or even has the opposite sign of the ITT effect. This contradicts the common assertion that the ITT effect is a conservative estimate of the total treatment effect [15]. Ignoring sampling variability, Balke and Pearl [5] derived nonparametric bounds for the total treatment effect under the DAG in Fig. 3 when all measured variables are binary, i.e., a range of values that are guaranteed to include the true value of the treatment effect. Other authors have developed parametric estimation methods for the total treatment effect, including two-stage estimation and G-estimation; see Hernan and Robins [16] and the references therein.

### Notation

To discuss the consequences of noncompliance, and relate this to non-blinding and drop-out, we introduce the variable *C*, which is the binary indicator of whether the subject complies with the (blinded or unblinded) protocol. That is, *C=1* if the subject takes what is assigned to them, *C=0* else. We note that, even if *C=0*, the subject may take the treatment that the protocol prescribes, although without being blind to it. For instance, if the subject is blindly assigned to placebo treatment (*Z=0*) and refuses to take it (*C=0*), and does not procure the active treatment by their own means, then *X=0* as the protocol prescribes. Similarly, if the subject is blindly assigned to active treatment (*Z=1*) and refuses to take it (*C=0*), and then procures the active treatment by their own means, then *X=1* as the protocol prescribes. Notably though, in such situations the treatment actually taken may be confounded with the outcome, even though it happens to be equal to the assigned treatment.

We allow for the possibility that *C* is measured, which would be the case in some trials. For example, in the case of a surgical treatment (for an unblinded trial), *C* would be determined by whether patients show up for their scheduled procedure. Other methods to measure *C* include counting the remaining pills in a treatment box or asking patients to self-report their level of compliance through a questionnaire. In some cases, researchers may also use electronic monitoring devices, such as a pill bottle cap that records when it is opened, to track whether participants comply with the treatment assignment.

Let *C*(*z*) be the compliance status *C* had, potentially counter to fact, the assigned treatment been set to *Z*. In the causal inference literature on RCTs with noncompliance, it is common to use the term ‘compliers’ for the principal stratum of subjects who would comply regardless of what treatment they were counterfactually assigned to; *C(0)=C(1)=1* [17]. Similarly, it is common to use the term ‘noncompliers’ for the principal stratum of subjects who would not comply regardless of what treatment they were counterfactually assigned to; *C(0)=C(1)=0*. We here use the terms ‘factual compliers’ and ‘factual noncompliers’, to refer to groups of subjects that factually complies, *C=1*, and factually does not comply, *C=0*, respectively. When the trial is blinded, *Z* has no effect on *C*, so that *C=C(0)=C(1)*. Hence, in this setting the factual (non)compliers and principal stratum (non)compliers are identical. However, this is not generally true when the trial is unblinded, since the compliance behavior may then depend on the assigned treatment.

### Blinded trial

**Fig. 4:**
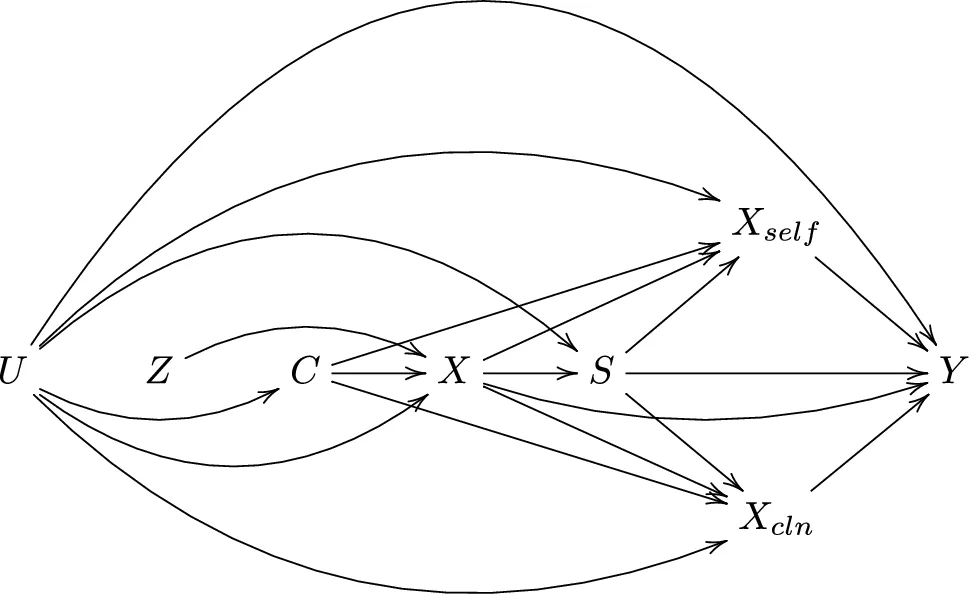
A blinded trial with noncompliance

The DAG in Fig. 4 represents a blinded trial with noncompliance but no drop-out. This DAG includes *Z*, as *Z* may not equal *X*. However, due to the blinding, *Z* has no effect on any of the variables in the DAG, except *X*. In general, the factors *U* that influence the outcome may also influence the decision to comply or not comply, as represented by the arrow from *U* to *C*, and may also influence the choice of treatment level for the factual noncompliers, as represented by the arrow from *U* to *X*. The arrow from *C* to *X* represents the special type of effect modification that, if *C=1* then *X=Z*, so that *U* has no effect on *X*, and if *C=0* then *Z* has no effect on *X*. The arrows from *C* to $X_{self}$ and $X_{cln}$ represents the special types of effect modification that, if *C=1* then *X* has no direct effect on $X_{self}$ and $X_{cln}$ not mediated through *S*, and if *C=0* then $X_{self}=X_{cln}=X$, so that *U* and *S* have no effect on $X_{self}$ and $X_{cln}$.

In this setting, only $\theta _{ITT}$ is nonparametrically identifiable, as *E(Y|Z=1)-E(Y|Z=0)*. However, the variable *Z* is a valid IV under the DAG in Fig. 4, and can be used to infer the presence of a treatment effect for some subjects, construct nonparametric bounds on $\theta _{trt}$, and, under additional parametric assumptions, estimate this effect, as outlined in "The ‘standard DAG’ for noncompliance" section. This is not true for the physiological treatment effect, which can be identically zero for all subjects, even when $\theta _{ITT} \ne 0$.

**Fig. 5:**
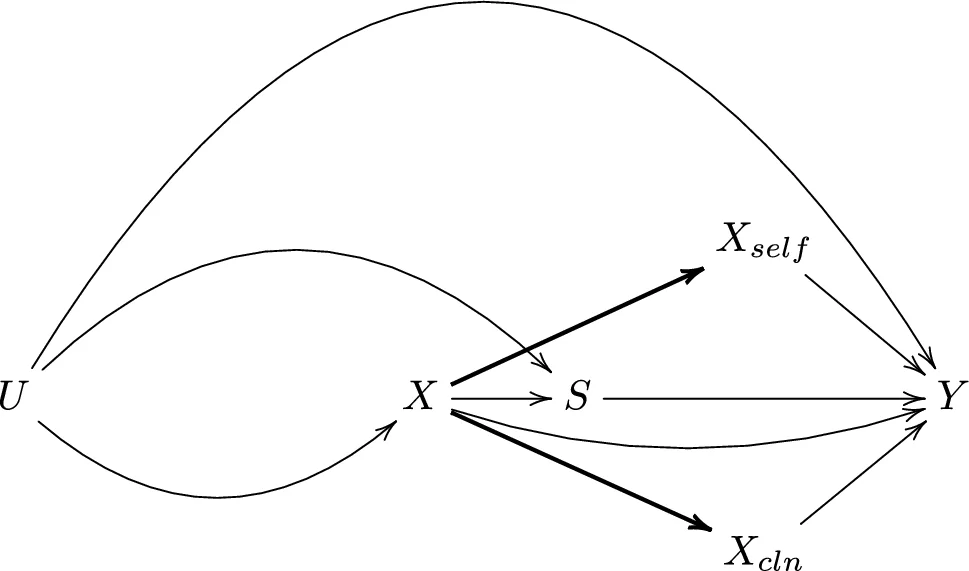
A blinded trial with noncompliance, for the subset of factual noncompliers

Since only *U* has an effect on *C* in Fig. 4, we can stratify on *C* (if *C* is measured) without inducing collider-stratification bias, and consider separately the subset of factual compliers (i.e., those with *C=1*) and factual noncompliers (i.e., those with *C=0*). For factual compliers, the trial can be represented with the DAG in Fig. 1, and for factual noncompliers, the DAG in Fig. 5 is representative. We have again used bold arrows to indicate the deterministic relations $X_{self}=X_{cln}=X$, in the DAG in Fig. 5.

Considering factual compliers and noncompliers separately, we define the conditional effects

$$\begin{aligned} \theta _{ITT\cdot c}=E\{Y(z=1)|C=c\}-E\{Y(z=0)|C=c\}, \\ \theta _{trt\cdot c}=E\{Y(x=1)|C=c\}-E\{Y(x=0)|C=c\} \end{aligned}$$

and

$$\begin{aligned} \theta _{phys\cdot c} & =E[Y\{x=1,X_{self}(0),X_{cln}(0)\}|C=c] \\ & \quad -E[Y\{x=0,X_{self}(0),X_{cln}(0)\}|C=c]. \end{aligned}$$

Among the factual compliers, we have that $\theta _{ITT\cdot 1}=\theta _{trt\cdot 1}$, and these two conditional estimands are nonparametrically identifiable as *E(Y|Z=1,C=1)-E(Y|Z=0,C=1)=E(Y|X=1,* *C=1)-E(Y|X=0,C=1).* Identification of $\theta _{phys\cdot c}$ requires additional assumptions, as outlined in "Perfect compliance and no drop-out" section. Among the factual noncompliers, only $\theta _{ITT\cdot 0}=E(Y|Z=1,C=0)-E(Y|Z=0,C=0)$ is nonparametrically identifiable.

Even though $\theta _{ITT.1}$, $\theta _{ITT.0}$ and $\theta _{trt.1}$ are identifiable, they may often be of limited clinical relevance. This is because factual compliance status *C* is not a ‘pre-treatment characteristic’, but a feature that becomes realized ones the treatment has been assigned. Thus, one cannot in realistic settings use effect measures conditional on *C* to, for instance, guide treatment decisions and tailor the treatment to patients that will benefit most from it.

### Unblinded trial

**Fig. 6:**
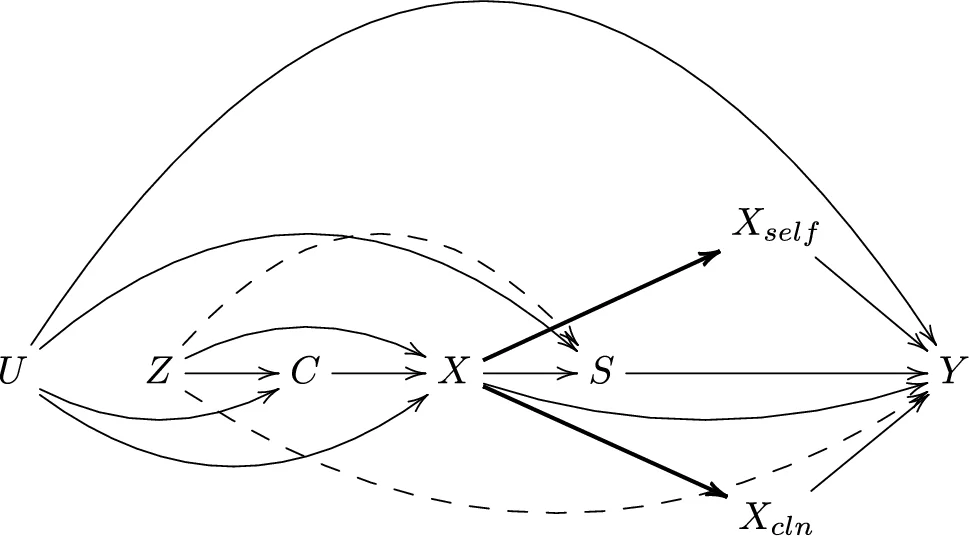
An unblinded trial with noncompliance

The DAG in Fig. 6 represents an unblinded trial with noncompliance, but no drop-out. We have explicitly included the assigned treatment, *Z*, as *X* may not equal *Z*. Just as in the unblinded trial with perfect compliance, the treatment believed by the subject and the clinician to have been taken equals the treatment actually taken, $X_{self}=X_{cln}=X$, as indicated by bold arrows.

The dashed arrows from *Z* to *S* and *Y* represent a direct effect of the assigned treatment on the intermediate health status during the trial and on the outcome at the end of the trial, respectively, not through the treatment actually taken. We have dashed these arrows since there are settings where they are arguably very weak, or entirely absent. This may, for instance, be the case in settings with hard clinical endpoints, such as death, where it is unlikely that a patient’s (feelings about) assigned treatment will have a direct effect on the outcome and the study protocol prevents follow-up difference by assignment. In such settings, *Z* is a valid IV and can be used to make inference on the total treatment effect, as outlined in "The ‘standard DAG’ for noncompliance" section. However, just as in the blinded setting with noncompliance, this does not provide a means of estimating the physiological treatment effect.

There are also settings where the effects along the dashed arrows could be present and non-negligible. For example, the trial team may plan differing follow-up by arm, regardless of compliance or treatment actually taken. A subject knowing they were randomized to receive no treatment may feel hopeless and depressed. This may be particularly true in life-threatening settings where the treatment is experimental and cannot be obtained outside the trial. Although we conceded the latter is potentially unlikely in a pragmatic study, the former is often true as resources are limited and sparing trial visits for patients on no treatment may allow for larger enrollment while not increasing costs.

In such settings, *Z* is not a valid IV, since the third IV assumption is violated by the causal paths from *Z* to *Y* that do not pass through *X*. We emphasize that this does not invalidate $\theta _{ITT}$ as such, which is still non-parametrically identifiable as *E(Y|Z=1)-E(Y|Z=0)*. However, it means that the ITT analysis is not informative about the effect of the treatment, neither the total effect nor the physiological effect, since a statistical association between *Z* and *Y* can then be partly or fully explained by the causal paths from *Z* to *Y* not through *X*.

We also note that, regardless of the existence of the dashed arrows, due to the pathway from *Z* to *C* the effects $\theta _{ITT\cdot 1}$ and $\theta _{trt\cdot 1}$ are no longer identifiable. Specifically, conditioning on *C=1* will make *Z* and *Y* statistically associated through the pathway $Z\rightarrow C\leftarrow U\rightarrow Y$ at which *C* is a collider, even in the absence of a causal effect of *Z* on *Y*.

## Trials with drop-out

The problems with drop-out are similar in trials with and without blinding, and in trials with and without noncompliance. Thus, for simplicity, we focus on the blinded trial with perfect compliance, and comment on the other trials at the end of the section. We assume that the consequence of drop-out is that the outcome is unobserved, but that all other variables that would normally be observed remain so. We let $O_{Y}$ be the indicator of observing the outcome, i.e., $O_{Y}=1$ if the subject does not drop-out so that the outcome is observed, $O_{Y}=0$ else.

**Fig. 7:**
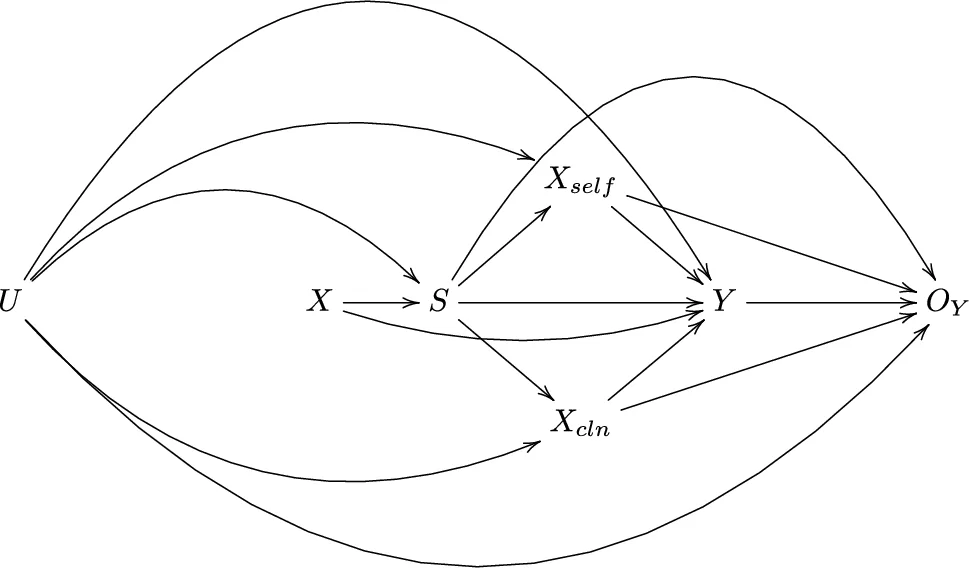
A blinded trial with perfect compliance and drop-out

The DAG in Fig. 7 represents a blinded trial with perfect compliance and drop-out. By including the indicator $O_Y$ of having observed the outcome, this DAG belongs to the class of m-graphs (‘m’ for ‘missing’), which have recently gained popularity in the missing data literature; see Mohan and Pearl [18] and the references therein. The DAG assumes a temporal ordering between *Y* and $O_Y$, such that *Y* ‘happens first’. Although we believe that this is a realistic model for many trials, we note that the opposite ordering could sometimes also make sense. For instance, a health outcome could become worse if a subject drops out, if the subject then no longer has access to the supportive environment and monitoring provided by the trial.

In Fig. 7, we have allowed for all variables to potentially affect $O_Y$, except *X* since it is difficult to imagine how the treatment taken could have a direct effect on the drop-out that does not pass through the intermediate health status or believes about what was taken. A direct effect of *S* on $O_{Y}$ would be present if side effects or lack of noticeable improvement during follow-up drive individuals to drop out of the trial. A direct effect of *Y* on $O_Y$ would be present if, for instance, individuals infer their value of *Y* before being measured by the clinician, and drop out due to fear or embarrassment about it being measured and recorded.

As in the blinded trial with perfect compliance and no drop-out, we have that $\theta _{ITT}=\theta _{trt}$, whereas $\theta _{phys}$ is generally different. However, under the DAG in Fig. 7, conditioning on having observed the outcome, $O_Y=1$, opens several non-causal paths between *X* and *Y*, on which $O_Y$ is a collider. Thus, restricting the analysis to those subjects for which the outcome is observed does not generally allow for the identification of any of the estimands of interest.

It is generally not possible to test the presence or absence of each specific arrow pointing into $O_Y$, since *U*, *S* $X_{self}$, and $X_{cln}$ are unmeasured. However, in some situations, we may still be able to construct a valid test for a treatment effect. Specifically, assuming that the observed data distribution is faithful to the DAG (i.e., that there are no other conditional independencies than those implied by the DAG; [19]), we can conclude that there is a treatment effect for *at least some subjects*, if we observe that the treatment taken is independent of the drop-out, $X{\perp \!\!\!\!\perp }O_Y$, and that the treatment taken is conditionally associated with the outcome, given that the outcome was observed, $X{\not \perp \!\!\!\!\perp }Y|O_Y=1$. To see this, note that the condition $X{\not \perp \!\!\!\!\perp }Y|O_Y=1$ implies that there is at least one causal path from *X* to *Y*, in which case there is a treatment effect for at least some subjects, or there is at least one non-causal path between *X* and *Y* that is open when conditioning on $O_Y=1$. However, all such non-causal paths in Fig. 7 include a causal path from *X* to $O_Y$, for instance, the non-causal path $X\rightarrow S\rightarrow O_Y \leftarrow Y$. Under faithfulness, the condition $X{\perp \!\!\!\!\perp }O_Y$ rules out all such causal paths from *X* to $O_Y$, and thus all non-causal paths between *X* and *Y* that are open when conditioning on $O_Y=1$.

Faithfulness may seem like a reasonable assumption, since unfaithfulness would require a perfect cancellation of different paths to produce exact independence between *X* and $O_Y$, which does not seem *a priori* likely. However, we note that, in any finite sample we would never observe an exact independence between *X* and $O_Y$ (e.g., an odds ratio exactly equal to 1 together with a p-value exactly equal to 1). Thus, to use the suggested test in practice, one would have to judge whether an observed association between *X* and $O_Y$ is ‘weak enough’ to warrant a causal conclusion, when also observing a conditional, hopefully strong, association between *X* and *Y*, given $O_Y=1$.

We can also construct bounds for $\theta _{ITT}$ (=$\theta _{trt}$), and/or conduct a sensitivity analysis, as follows. Define the ‘observed ITT effect’

$$\begin{aligned} \theta _{ITT}^{obs}=E(Y|Z=1,O_Y=1)-E(Y|Z=0,O_Y=1), \end{aligned}$$

and the parameters $\Delta (x)=E(Y|X=x,O_Y=0)-E(Y|X=x,O_Y=1)$ and $p(x)=p(O_Y=1|X=x)$ for *x=0,1*. The parameters *Δ (0)* and *Δ (1)* are not generally identifiable, and measure the degree of deviation from the ideal setting where *Y* is conditionally independent of $O_Y$, given *X*. In this setting, $\Delta (0)=\Delta (1)=0$ and $\theta _{ITT}=\theta _{ITT}^{obs}$. It is easy to show that

$$\begin{aligned} \theta _{ITT}=\theta _{ITT}^{obs}+\Delta (1)\{1-p(1)\}-\Delta (0)\{1-p(0)\}. \end{aligned}$$

By varying *Δ (1)* and *Δ (0)* in this expression over a range of plausible values, one obtains a range of plausible values for $\theta _{ITT}$. If *Y* is binary, then $-E(Y|X=x,O_Y=1)\le \Delta (x)\le 1-E(Y|X=x,O_Y=1)$, so that taking *Δ (0)* and *Δ (1)* to their extreme values in this sensitivity analysis gives the nonparametric bounds

1 $$\begin{aligned} \theta _{ITT}^{obs} & -E(Y|X=1,O_Y=1)\{1-p(1)\} \\ & -\{1-E(Y|X=0,O_Y=1)\}\{1-p(0)\}\nonumber \\ & \quad \le \theta _{ITT}=\theta _{trt}\le \nonumber \\ \theta _{ITT}^{obs} & +\{1-E(Y|X=1,O_Y=1)\}\{1-p(1)\} \\ &+E(Y|X=0,O_Y=1)\{1-p(0)\} \end{aligned}$$

These bounds were derived by Gabriel at al. [20] (their equation (3)). As the degree of drop-out goes to 0, *p*(0) and *p*(1) go to 1, so that these bounds collapse into $\theta _{ITT}^{obs}$.

Analogous sensitivity analysis and bounds can be used for all parameters that are identifiable in the absence of drop-out, in the other settings discussed above, i.e., for $\theta _{ITT}=\theta _{trt}$ in unblinded trials with perfect compliance, for $\theta _{ITT\cdot 1}=\theta _{trt\cdot 1}$, $\theta _{ITT\cdot 0}$ and $\theta _{ITT}$ in blinded trials with noncompliance, and for $\theta _{ITT}$ in unblinded trials with noncompliance. Although it is possible to construct partial-identification bounds or carry out a sensitivity analysis for $\theta _{phys}$, this is more involved and likely to not exclude the causal null, since $\theta _{phys}$ is not identifiable in any of the settings that we have considered, even without drop-out.

The bounds in (1) were proven by Gabriel et al. [20] to be sharp when one makes no assumptions about which variables do and do not affect $O_Y$, i.e., when all arrows pointing to $O_Y$ in Fig. 7 are potentially present. In some settings, one may be confident that some of these arrows are absent, e.g., that *X* has no direct effect on whether *Y* is observed, not mediated through *Y*. Gabriel et al. [20] derived bounds on $\theta _{ITT}$ and $\theta _{trt}$ that are potentially narrower than those in (1), by exploiting such assumptions.

## Discussion

We have used DAGs to discuss how unblinding, noncompliance, and drop-out may hamper the identifiability of three possible estimands; the ITT effect, the total treatment effect, and the physiological treatment effect. We have highlighted that, in pragmatic trial settings without blinding, the randomly assigned treatment may not be a valid IV for the treatment actually taken, and thus, neither the total treatment effect nor the physiological treatment effect can be tested to differ from zero. We have shown how one can conduct sensitivity analysis and construct bounds when there is drop-out.

We have focused on point outcomes that are not censored. In our experience, most pragmatic studies will either have the same follow-up for all subjects, for example, via death registers, or use some type of score or biomarker as the outcome. Thus, we do not see our focus as greatly limiting the applicability of our settings or discussion. For time-to-event outcomes, one would need to redefine the estimands as, for instance, counterfactual survival differences. Informative censoring of time-to-event outcomes is similar to informative drop-out, and can be handled with similar methods as those that we have discussed, e.g., sensitivity analyses and bounds.

We have also only considered point treatments, which we concede limits our DAGs applicability. Although similar arguments and DAGs can be used within periods of a trial, one would expect daily treatment, for example, to also increase the complexity of the relationships, as previous treatment potentially impacts compliance later in the study. Thus, an extension to treatments taken multiple times over the trial period would be valuable but much more complex.

The main obstacle to identifiability of the total treatment effect in the presence of noncompliance is the presence of confounders for the treatment actually taken and the outcome, represented by *U* in Figs. 3–6. If these confounders were entirely measured, then the total treatment would be identifiable, provided that there is no drop-out. In the presence of drop-out, complete measurement of these confounders does not make the total treatment effect (or the ITT effect) identifiable, since the outcome remains unobserved for a subgroup of subjects.

Identification of the physiological treatment effect is more difficult. In particular, $\theta _{phys}$ is non-identifiable in a blinded trial with perfect compliance and no drop-out (Fig. 1), without additional assumptions not implied by the design, even if all variables on the DAG were measured, including the confounders *U*, the intermediate health status *S*, and the subject’s and clinicians’ beliefs $X_{self}$ and $X_{cln}$. This is because the variable *S* is a ‘recanting witness’ under the DAG in Fig. 1, in the sense that it is part of both the natural direct effect $\theta _{phys}$ through the path $X\rightarrow S\rightarrow Y$, and the corresponding natural indirect effects through the paths $X\rightarrow S\rightarrow X_{self}\rightarrow Y$ and $X\rightarrow S\rightarrow X_{cln}\rightarrow Y$. It has been shown that the presence of a recanting witness hampers the identification of natural direct effects [21].

Although we believe the physiological treatment effect may often be of greater scientific interest than the total treatment effect and the ITT effect, we do not mean to imply that one should ignore issues of nonidentifiability when defining the estimand. Rather, we agree with Vansteelandt and Van Lancker [22] in that ‘when selecting a suitable causal estimand, striking a balance becomes imperative between the right question and the feasibility to answer it under realistic assumptions’. In particular, if the trial that one is planning for will not realistically come close to identifying the physiological treatment effect, then it is somewhat pointless to specify this as the target estimand of that trial. However, we also note that identification is not a clear-cut dichotomy; sometimes, one may be able to carry out a sensitivity analysis or derive partial-identification bounds for an estimand that is not point-identifiable under the assumptions one is willing to make.

We have defined the physiological treatment effect as a natural direct effect, but one could also define it as a controlled direct effect, i.e., as some contrast between $E\{Y(x=1,x_{self},x_{cln})\}$ and $E\{Y(x=0,x_{self},x_{cln})\}$ for fixed values $x_{self}$ and $x_{cln}$. This is essentially how Stensrud et al. [23] defined the immunologic effect (their equation (3)) in the context of vaccine trials, to distinguish it from the behavioral (e.g., non-physiological) effect (their equation (4)). Some may find the controlled direct effect more appealing than the natural direct effect, since it does not require nested counterfactuals of the form $Y\{x,X_{self}(x'),X_{cln}(x')\}$. However, the controlled direct effect requires assumptions/conditions for identifiability that are different, and not necessarily weaker, than those required by the natural direct effect. In particular, as discussed in "Blinded trial" section, the natural direct effect is identifiable—and equal to the total treatment effect—in a blinded trial with perfect compliance and no drop-out, under the additional assumption that the blinding entirely removes the influence of the treatment actually taken *X* on the subject’s and clinician’s believes $X_{self}$ and $X_{cln}$ (so that *S* is not a recanting witness). However, this does not make the controlled direct effect identifiable. Instead, one way to identify the controlled direct effect would be to measure $X_{self}$ and $X_{cln}$ and explicitly condition on these in the analysis. One would then also have to measure and condition on the whole set of confounders *U*, or simply assume that these confounders are entirely absent, to avoid bias due to mediator-outcome confounding. This essentially corresponds to the type of trial that Stensrud et al. [23] denoted ‘$\tau _{II}$’. Alternatively, one could attempt to control $X_{self}$ and $X_{cln}$ by design, e.g., by telling all subjects, possibly contrary to fact, that they received active treatment. Stensrud et al. [23] denoted this type of trial with ‘$\tau _{IV}$’. Clearly, such $\tau _{II}$ and $\tau _{IV}$ trials are quite hypothetical, and we conjecture that the controlled direct effect is often more difficult to identify in practice than the natural direct effect in blinded randomized trials.

In each setting, we have considered nonparametric identification under a ‘worst-case scenario’, in the sense that our DAGs have included all arrows that are not ruled out by design of the trial. In reality, the analyst may be willing to assume that certain arrows are entirely absent, or that (the effects along) some paths in the DAG are negligible relative to the other paths. Furthermore, the analyst may be willing to make parametric model assumptions, for instance, when using the assigned treatment as an IV in two-stage estimation or G-estimation. Such assumptions may lead to stronger identifiability results than those we have presented. However, as the assumptions that may apply in a given setting will be highly context-dependent, we have chosen not to elaborate further.

Recently, SWIGs have become a popular alternative to DAGs [24]. In the Supplementary Material, we provide SWIGs for each scenario that we have considered. We also use these SWIGs to provide an alternative definition of the physiological treatment effect, based on the notation of ‘separable effects’ [25].

Although we have defined $\theta _{ITT}$, $\theta _{trt}$ and $\theta _{phys}$ unconditionally, there is an implicit conditioning on being included in the trial. This may raise concerns about transportability of these effects, e.g., if the trial used selective inclusion criteria, or if participation in the trial has effects on the outcome, so-called ‘trial engagement effects’. We refer to Dahabreh and Hernan [26] and Ung et al. [27], and the references therein, for discussions of such transportability issues.

In conclusion, we hope this paper will serve as a valuable resource for researchers planning to conduct an RCT and writing a Statistical Analysis Plan (SAP). It emphasizes the distinctions between the ITT effect, total treatment effect, and physiological treatment effect, while highlighting how common trial imperfections can hinder the identifiability of these estimands. Additionally, we discuss the assumptions necessary to achieve identifiability. Thus, if a trial statistician considers identification in the SAP under the worst-case-scenario DAGs provided herein, they not only know if their estimand of interest will be estimable with these complications, they also know what complications need to be mitigated in practice to reduce bias.

## Supplementary Information

Below is the link to the electronic supplementary material.

Supplementary file1 (PDF 175 kb)
