# Supplementary material for: Elucidating some common biases in randomized controlled trials using directed acyclic graphs
Source: Eur J Epidemiol. 2025 Sep 11;41(6):697–707. doi: 10.1007/s10654-025-01298-7 (PMC13424470; doi:10.1007/s10654-025-01298-7)
Supplement: Supplementary file 1 [file 10654_2025_1298_MOESM1_ESM.pdf]

Supplementary materials for "Elucidating Some Common Biases in Randomized Controlled Trials using Directed Acyclic Graphs"

Erin E Gabriel[1*†], Alex Ocampo[2] and Arvid Sjölander[3†]

[1*]Section of Biostatistics, Department of Public Health University of Copenhagen Copenhagen, Denmark.
[2]Novartis Pharma AG, Basel, Switzerland.
[3]Department of Medical Epidemiology and Biostatistics, Karolinska Institutet, Sweden.

*Corresponding author(s). E-mail(s): erin.gabriel@sund.ku.dk;
Contributing authors: arvid.sjolander@ku.se; alex.ocampo@novartis.com;
[†]These authors contributed equally to this work.

1 Single-world Intervention Graphs

Single-world intervention Graphs (SWIGs), as introduced by Robins and Richardson [1], unify the graphical and potential outcome approaches to causal inference. For each DAG in the main body of the paper, we draw a corresponding SWIG, which can be used to visualize the necessary identification assumptions required for identification of the estimands.

In brief, SWIGs are similar to DAGs but there are two key distinguishing factors. The first, and most important, is that nodes are split to denote interventions on random variables. For instance, if treatment X is split and set to $x = 1$, then we are envisaging a world in which everyone in the population took treatment $x = 1$. The splitting of the node corresponds to the "what if?" question of the causal estimand - e.g. what would the potential outcomes be if everyone took active treatment (or placebo). The second distinction follows from the first, which is that all variables downstream of those intervened on become potential outcomes. For a tutorial, see [2].

1.1 SWIGs for ITT estimands

Recall the the ITT estimand:

$$\theta_{ITT} = E\{Y(z=1)\} - E\{Y(z=0)\},$$

To depict the ITT estimand in a SWIG, we split the node of Z on the DAG to represent an intervention on randomized treatment assignment. This "hypothetical" intervention is exactly what the causal ITT estimand postulates - i.e. what if we had set randomized treatment to 1 (or analogously 0) for all patients?

Note that for trials with perfect compliance $X = Z$ and so we replace X on the DAG with Z on the SWIG in order to be specific about the intervention postulated that defines the causal effect of interest. Below are SWIGs for all the DAGs presented in the main body of the paper with $Z = z$.

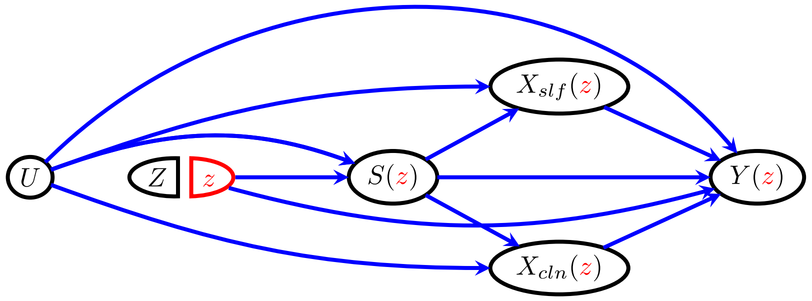

Fig. 1: SWIG for a blinded trial with perfect compliance

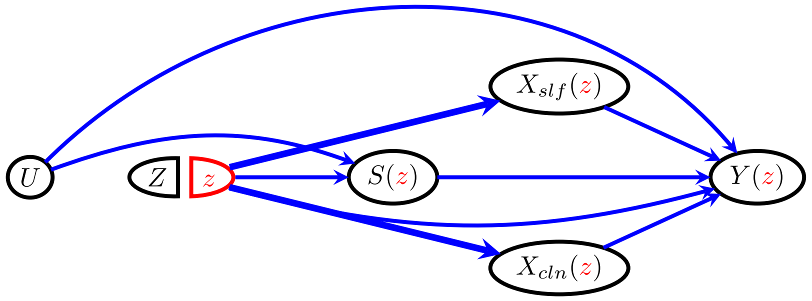

Fig. 2: SWIG for an unblinded trial with perfect compliance

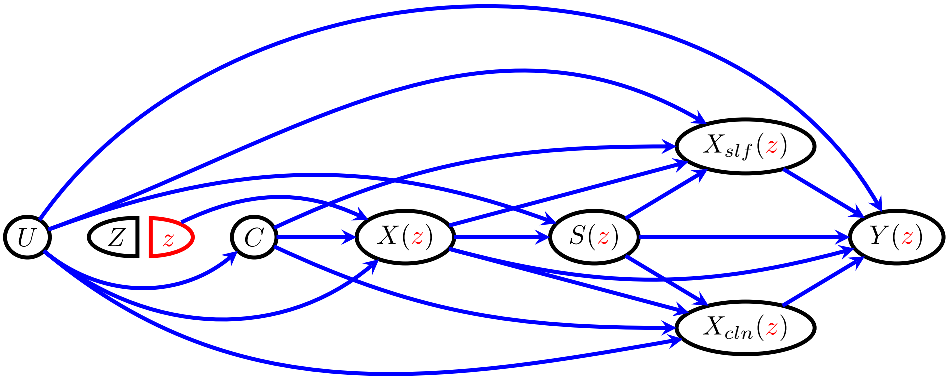

Fig. 3: SWIG for a blinded trial with noncompliance

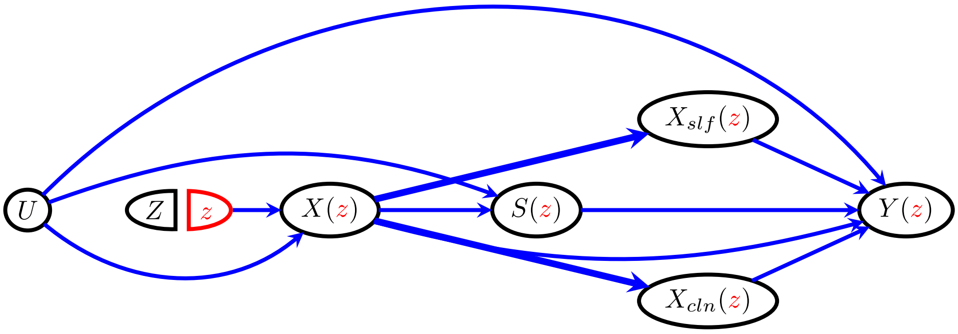

Fig. 4: SWIG for an blinded trial with noncompliance, for the subset of factual non-compliers

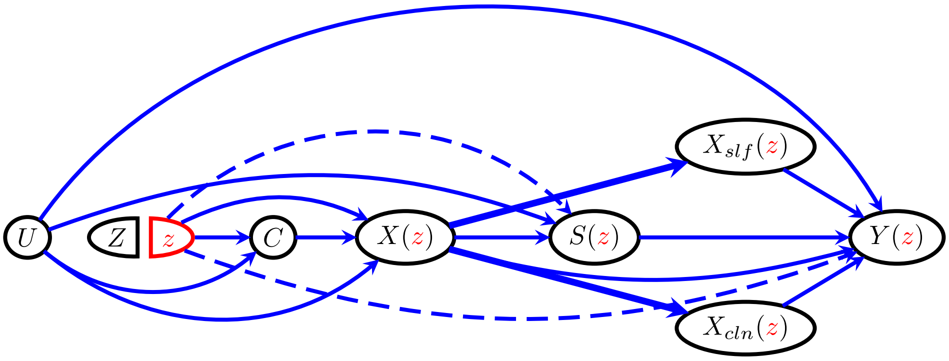

Fig. 5: SWIG for an unblinded trial with noncompliance

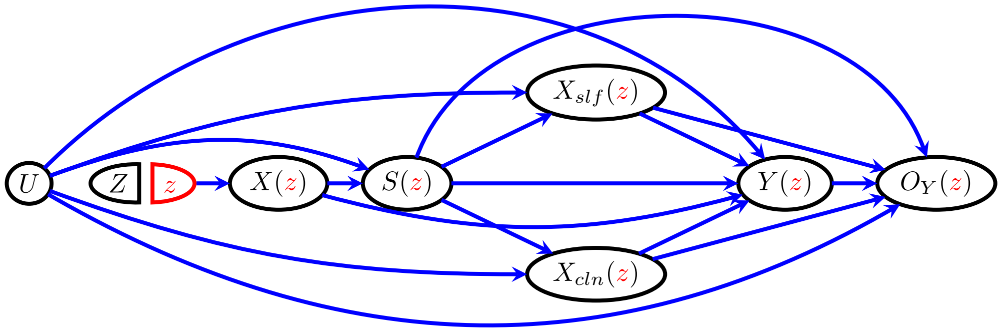

Fig. 6: SWIG for an blinded trial with perfect compliance and dropout

Identification of the ITT estimand is generally simple since $Z \perp Y(z)$ due to randomization, but that is not the case in the case with dropout, where we condition on O_Y.

1.2 SWIGs for the total treatment effect

Next, we consider SWIGs for the total treatment effect estimand:

$$\theta_{trt} = E\{Y(x=1)\} - E\{Y(x=0)\}$$

In contrast to the SWIGs above in section 1.1, which split the Z variable node, SWIGs for the total treatment effect will split the X node. This is clearly seen in the potential outcome definition of the estimand, which considers $Y(x)$ for $x \in 0,1$. That is, the total treatment effect considers an intervention on the actual treatment taken, not just the treatment assigned. As outlined in the main text, identification of the total treatment effect is more difficult, which can be seen from the backdoor paths in many of these SWIGs between X and $Y(x)$. Additionally, as with the ITT effect, although there are no backdoor paths from X to Y in Figure 12, due to conditioning on O_Y, the population effect cannot be identified.

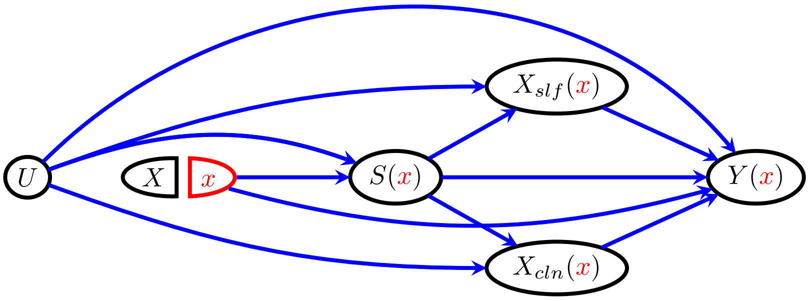

Fig. 7: SWIG for a blinded trial with perfect compliance

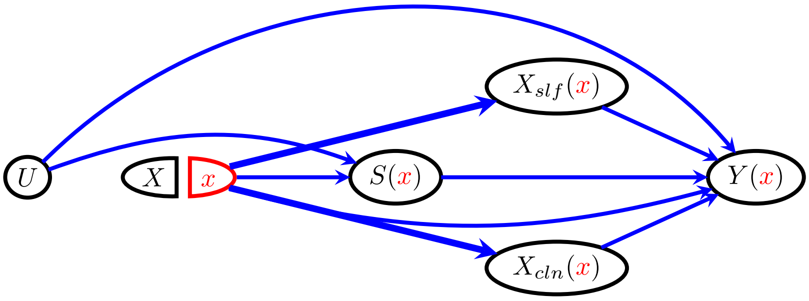

Fig. 8: SWIG for an unblinded trial with perfect compliance

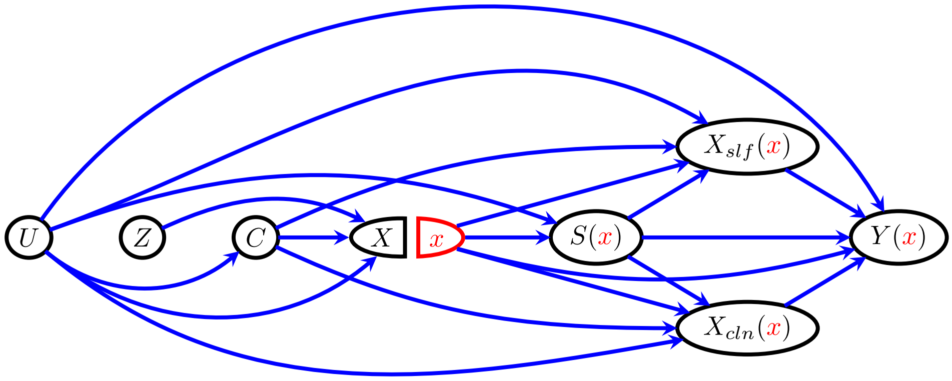

Fig. 9: SWIG for a blinded trial with noncompliance

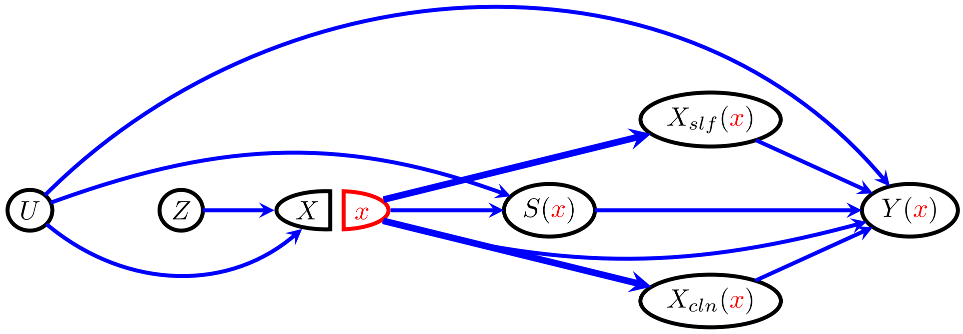

Fig. 10: SWIG for an blinded trial with noncompliance, for the subset of factual noncompliers

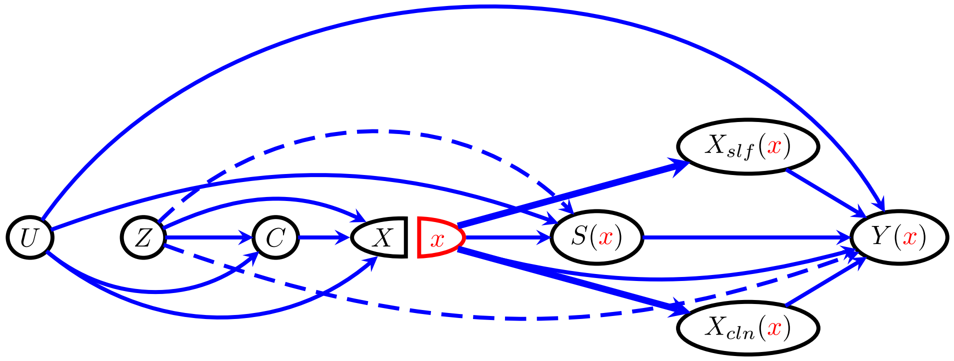

Fig. 11: SWIG for an unblinded trial with noncompliance

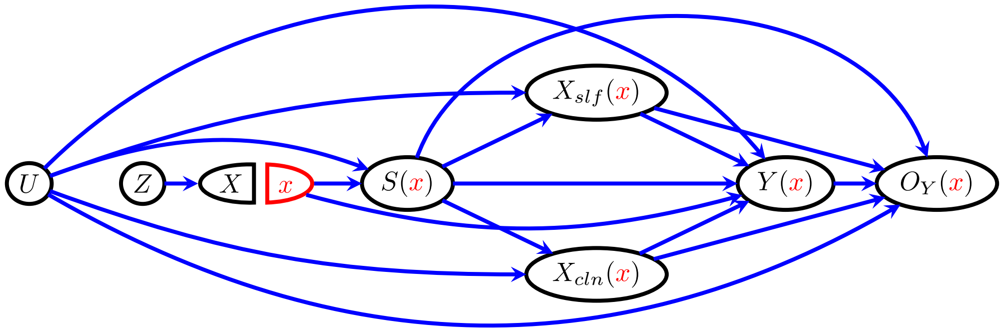

Fig. 12: SWIG for an blinded trial with perfect compliance and dropout

1.3 SWIGs for the physiological treatment effect

The most complex scenario to consider is that of the physiological treatment effect, defined as:

$$\begin{aligned}\theta_{phys} &= E[Y\{x=1, X_{self}(x=0), X_{cln}(x=0)\}] \\ &- E[Y\{x=0, X_{self}(x=0), X_{cln}(x=0)\}].\end{aligned}$$

This is defined as a natural direct effect. These types of mediation estimands are nested potential outcomes and cannot be represented in a SWIG due to their cross-world nature. It would require two SWIGs representing the two worlds and then making connections between them.

However, the separable effects framework allows similar estimands (and equivalent in terms of the numerical value estimated under certain conditions) to be depicted in a SWIG. Separable effects conceptualize an experiment that could (at least in theory) be conducted to measure the mediation effects. They are therefore empirically verifiable, unlike the cross-world nested counterfactuals that define the natural effects.

Blinding lends itself nicely to the separable effects framework, because we can envisage a world in which the treatment was either blinded or not, yet the physiological mechanism of the treatment remain the same. We will depict this in SWIGs.

As the blinding is part of the treatment assignment mechanism, the effect of blinding will flow out from the treatment assignment Z while the physiological effect from the actual physical treatment X. To define the separable effect, consider that the effect of treatment assignment Z exerts its downstream effect in two main ways. This means that the intervention has two assigned components, which are:

1. The actual medicinal treatment given X
2. The medicinal treatment patients are told they are taking Z^b

We use the superscript b on Z to correspond with "blinding". That is, when treatment is assigned, in addition to giving a particular medicine, the study coordinator also sets Z^b to one of the following:

$$Z^b = \begin{cases} 0 & \text{if patients and clinicians are told the patient is taking Placebo} \\ 1 & \text{if patients and clinicians are told the patient is taking Treatment} \\ NA & \text{if patients and clinicians are blinded} \end{cases}$$

In general clinical practice, $X = Z^b$ for all patients. However, we can now envisage scenarios (or experiments, or worlds) in which a patient is randomized to a particular treatment $X = x$ and also told by the investigators they are taking $Z^b = z^b$. In this thought experiment, it is possible that $z^b \neq x$, which would mean patient's and clinicians are intentionally being deceived. This may not be ethical depending on the context, but has been suggested in the literature to investigate the placebo response [3]. The fact that treatment assignment Z can be split into two pathways (X and Z^b)

is what makes it "separable". Therefore we can define the physiological effect as a separable direct effect with:

$$\theta_{phys} = E[Y(x = 1, z^b = 0)] - E[Y(x = 0, z^b = 0)]$$

This estimand aims to remove the psychological effect of treatment by telling all patients that they are on placebo, even if they are on active treatment. While this is not actually done in the study at hand, it is at least conceivable that such an experiment could be done, which is not necessarily the case for cross-world natural effects.

Specific to our case, one may need to make one further assumption, to avoid violation of the dismissible component conditions of separable effects: that patients and clinicians believe what the investigator has told them about their treatment taken. This would remove the arrows from $S(x)$ to $X_{slf}(x, z^b = 0)$ and $X_{cln}(x, z^b = 0)$. We do not necessarily make this assumption on any of the SWIGs drawn, but should it hold true then:

$$\begin{aligned}\theta_{phys} &= E[Y(x = 1, z^b = 0)] - E[Y(x = 0, z^b = 0)] \\ &= E[Y(x = 1, X_{self}(z^b = 0), X_{cln}(z^b = 0)] - E[Y(x = 0, X_{self}(z^b = 0), X_{cln}(z^b = 0)] \\ &= E[Y(x = 1, x_{self} = 0, x_{cln} = 0)] - E[Y(x = 0, x_{self} = 0, x_{cln} = 0)]\end{aligned}$$

Then this is consistent with a controlled direct effect. The last equality holds because setting z_b deterministically fixes $X_{slf} = z^b$ if the patient and clinician beleive what they are being told. By holding x_{self} and x_{cln} constant across both terms, this estimand measures purely the effect of treatment due only to the physiological changes caused by the treatment and separates this from the psychological effect knowing the treatment assignment. However, for the same reasons the natural direct effects are not identifiable, so do the separable effects fail to be identifiable in any setting we consider.

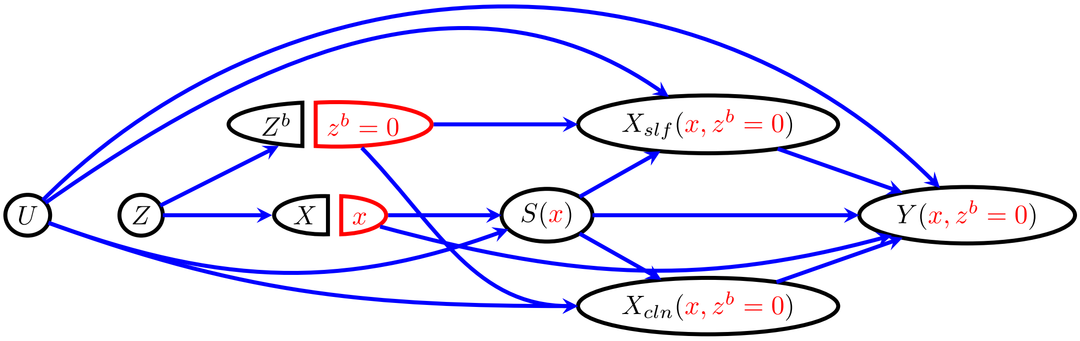

Fig. 13: SWIG for a blinded trial with perfect compliance

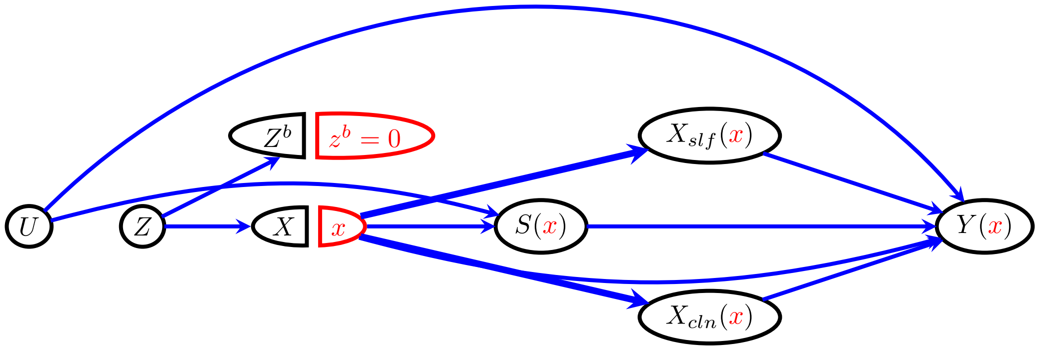

Fig. 14: SWIG for an unblinded trial with perfect compliance

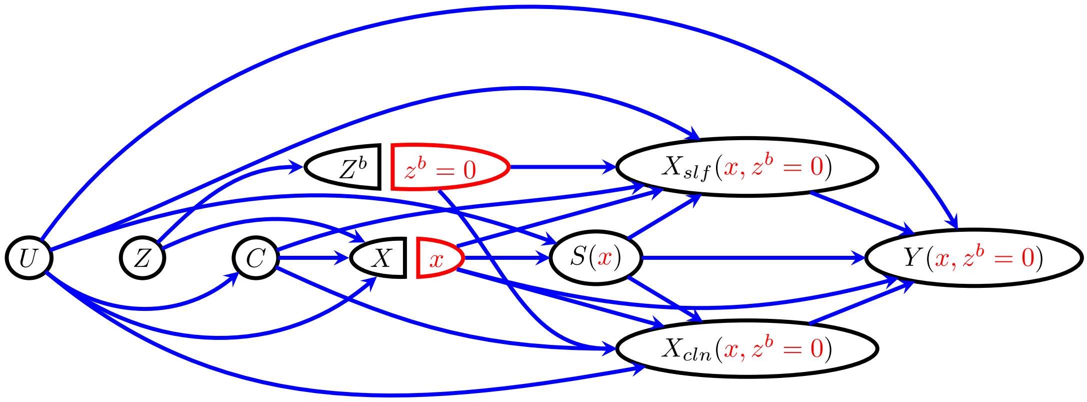

Fig. 15: SWIG for a blinded trial with noncompliance

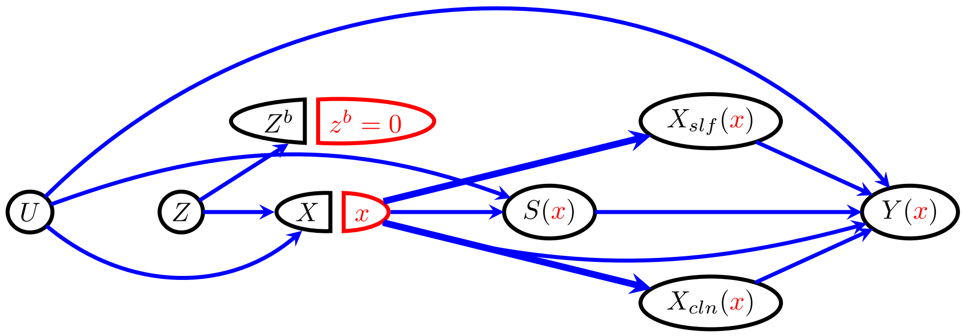

Fig. 16: SWIG for an blinded trial with noncompliance, for the subset of factual noncompliers

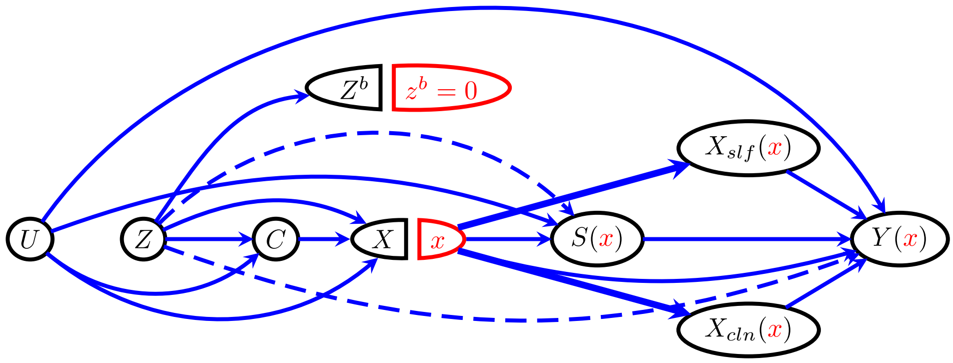

Fig. 17: SWIG for an unblinded trial with noncompliance

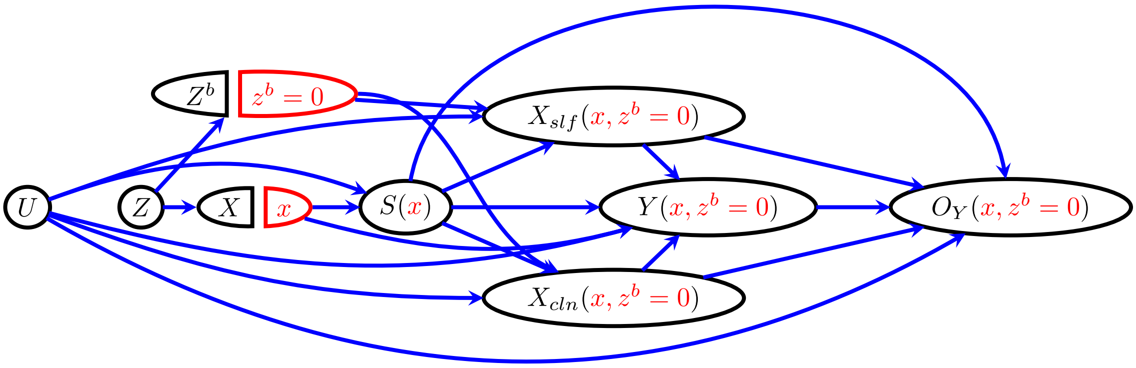

Fig. 18: SWIG for an blinded trial with perfect compliance and dropout

References

[1] Richardson, T.S., Robins, J.M.: Single world intervention graphs (swigs): A unification of the counterfactual and graphical approaches to causality. Center for the Statistics and the Social Sciences, University of Washington Series. Working Paper **128**(30), 2013 (2013)

[2] Ocampo, A., Bather, J.R.: Single-world intervention graphs for defining, identifying, and communicating estimands in clinical trials. Statistics in Medicine **42**(21), 3892–3902 (2023)

[3] Enck, P., Klosterhalfen, S., Zipfel, S.: Novel study designs to investigate the placebo response. BMC medical research methodology **11**, 1–8 (2011)
